# Pre-symptomatic detection of wheat stem rust using hyperspectral imaging and deep learning

**DOI:** 10.3389/fpls.2026.1873364

**Published:** 2026-06-19

**Authors:** Zhaowei Zhu, Min Lin, Chengjun Li, Jie Deng, Ning Ji

**Affiliations:** 1 School of Information and Intelligence Engineering, Tianjin Renai College, Tianjin, China; 2 College of Information and Electrical Engineering, China Agricultural University, Beijing, China; 3 School of Civil Engineering, Tianjin Renai College, Tianjin, China; 4 School of Mechanical and Power Engineering, Tianjin Renai College, Tianjin, China; 5 College of Water Resources and Civil Engineering, China Agricultural University, Beijing, China; 6 State Key Laboratory of Engines, Tianjin University, Tianjin, China

**Keywords:** deep learning, early detection, hyperspectral imaging, pre-symptomatic stage, wheat stem rust

## Abstract

**Introduction:**

Wheat stem rust (Puccinia graminis f. sp. tritici) remains a major threat to wheat production worldwide. Detecting the disease at the pre-symptomatic stage is important for earlier warning and more timely management.

**Methods:**

We evaluated hyperspectral imaging and deep learning for pre-symptomatic wheat stem rust detection using a time-series dataset collected at 4–9 days post inoculation (DPI 4–9). Seven representative deep learning models were compared across DPI stages. A weighted cross-entropy strategy was then applied to the three strongest models, and model interpretability was examined using input gradient analysis, SHAP attribution, and vegetation-index screening.

**Results:**

The weighted optimization increased overall F1-scores by 10.0%–18.4%. At the pre-symptomatic stage, the best model achieved an F1-score of 0.94 at DPI 4 and 0.99 at DPI 5, enabling detection before visible symptom development at DPI 6–7. Across the interpretability analyses, the 480–550 nm blue–green region emerged as the main source of information for pre-symptomatic detection, whereas the 750–870 nm near-infrared region contributed more general information on disease presence.

**Discussion:**

These results show that hyperspectral imaging paired with deep learning can support accurate pre-symptomatic detection of wheat stem rust under controlled experimental conditions and provide useful evidence for future field-scale studies of early disease warning.

## 1 Introduction

Plant diseases remain a major constraint on global food security, accounting for an estimated 20%–40% reduction in crop production each year (Savary et al., 2019). Wheat is among the world’s most important food crops, covering more than 217 million hectares and supplying roughly 20% of the calories and protein consumed globally (Shiferaw et al., 2013). Wheat stem rust, caused by *Puccinia graminis* f. sp. *tritici*, is one of the most destructive diseases affecting wheat production. Under favorable conditions, yield losses can reach 50%–100% (Soko et al., 2018; Rehman et al., 2024). The continued spread of highly virulent races represented by the Ug99 lineage has placed nearly 90% of commercial wheat cultivars at potential risk and has further increased the vulnerability of wheat production systems (Singh et al., 2015; Lewis et al., 2024). Against this background, earlier monitoring and faster response have become pressing needs.

Pre-symptomatic detection, that is, the identification of disease during the incubation period before visible symptoms appear, is central to earlier warning and more precise disease management. In wheat stem rust, the latent period is shaped by temperature, humidity, and host physiological status, and uredinia usually form about 8–10 days after infection under field conditions (Leonard and Szabo, 2005). This leaves only a limited window for intervention. During the pre-symptomatic stage, however, plants show no obvious visual symptoms; instead, infection is reflected mainly in weak physiological disturbance and subtle spectral change. Such signals are difficult to capture with conventional scouting. Current monitoring still relies largely on field inspection, which often identifies disease only after expansion is already under way (Sankaran et al., 2010). More sensitive sensing and recognition methods are therefore needed for this stage.

Hyperspectral imaging acquires spatial and continuous spectral information at the same time and does so in a non-destructive, label-free manner. This makes it well suited to studying the subtle physiological changes induced by pathogen infection before symptoms are visible (Lu et al., 2018; Mahlein et al., 2018; Hong et al., 2026). In recent years, hyperspectral imaging has been used successfully for disease detection, classification, and quantitative inversion in several crop systems (Deng et al., 2023b; Deng et al., 2023a; García-Vera et al., 2024). Relative to conventional RGB imaging, hyperspectral data can capture early responses linked to chlorophyll content, cellular structure, and water status, which makes earlier disease detection plausible (Shafay et al., 2025).

Deep learning is also relevant in this context because it can learn discriminative representations directly from hyperspectral data. Early disease detection from hyperspectral data has already been explored in several crop systems, including asymptomatic grapevine viral disease, latent rice bacterial leaf blight, and tomato spotted wilt virus in sweet pepper (Wang et al., 2019; Nguyen et al., 2021; Cao et al., 2022).

For wheat diseases, however, most existing studies still focus on stages at which visible symptoms are already present. Early symptomatic detection has been reported for wheat powdery mildew using hyperspectral vegetation indices and texture features with conventional machine learning and for stripe rust using field RGB images with ResNet-18 (Khan et al., 2021; Schirrmann et al., 2021). More recently, a time-series indoor hyperspectral dataset of wheat stem rust was analyzed with a systematic preprocessing pipeline and traditional machine learning, which increased the F1-score from 0.67–0.75 to 0.86–0.94 and enabled detection of asymptomatic infection at DPI 4 (Fedotov et al., 2025). That study nevertheless did not examine in a systematic way how deep learning performs for pre-symptomatic detection, nor did it compare the spectral cues emphasized by deep learning with those used in traditional feature engineering.

This study evaluates the potential of deep learning for pre-symptomatic detection of wheat stem rust. We focus on three questions. First, can deep learning models recognize wheat stem rust before visible symptoms appear? Second, how much earlier than human visual inspection can they provide useful detection, as judged from day-by-day analysis across days post inoculation (DPI)? Third, what spectral information do the models rely on in making these predictions? To address these questions, we built a systematic evaluation framework on the basis of a time-series hyperspectral dataset of wheat stem rust, compared seven representative deep learning models, and then optimized the strongest models with a weighted cross-entropy strategy. We further examined model behavior using input gradient analysis, SHAP attribution, and vegetation-index screening. Through this combined analysis of performance, timing, and spectral evidence, the study clarifies both the promise and the practical limits of hyperspectral deep learning for pre-symptomatic disease detection in crops.

## 2 Materials and methods

**Figure 1:**
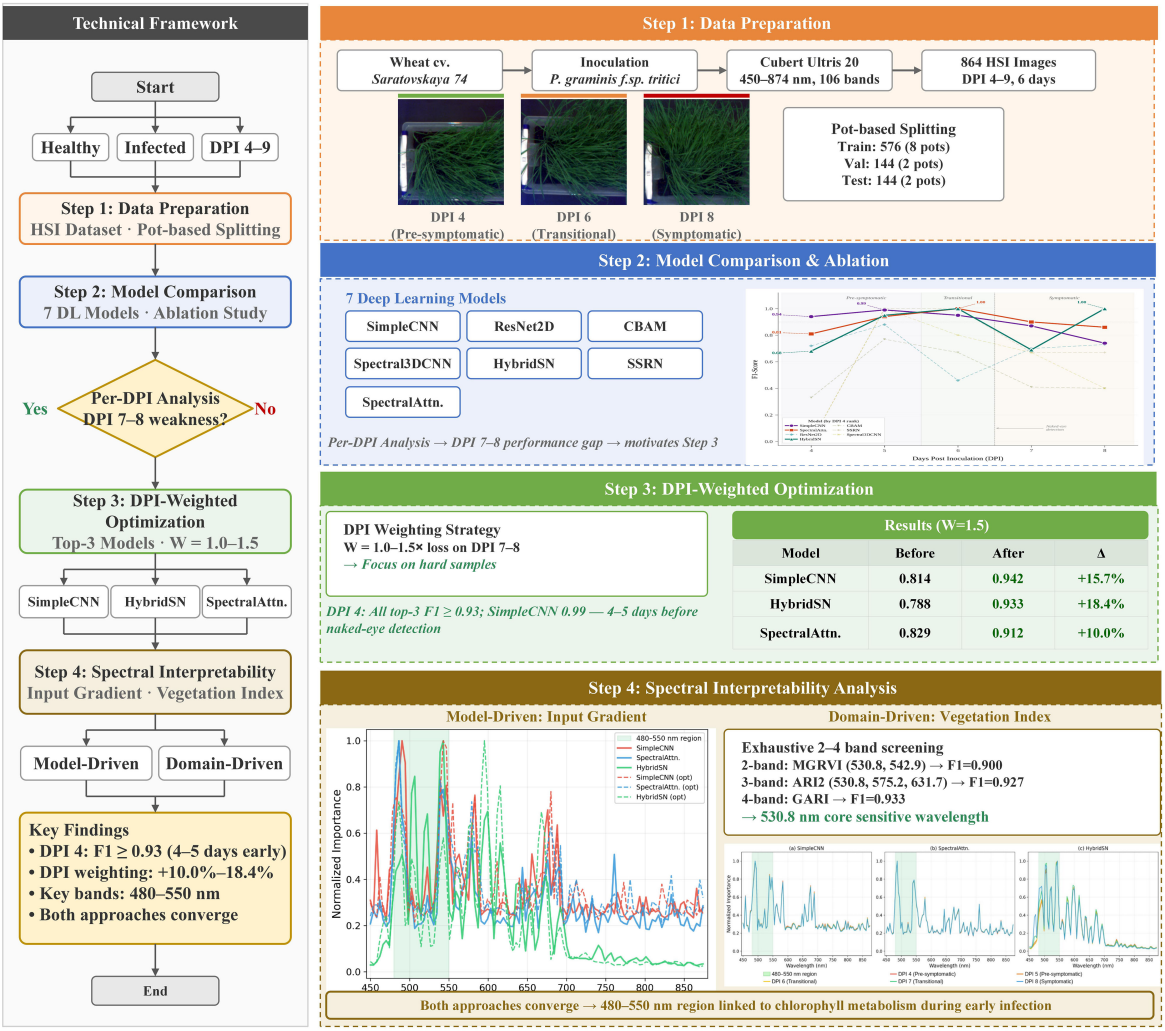
Overall workflow of the study. The workflow consisted of four stages: data preparation and pot-level partitioning; comparison of seven deep learning models and preprocessing ablation; DPI-weighted optimization of the three strongest models; and spectral interpretability analysis using input gradients, SHAP attribution, and vegetation-index screening.

The overall workflow of the study is shown in **Figure 1**. Briefly, the study was organized into four sequential stages: data preparation and pot-level partitioning, systematic comparison of seven deep learning models with preprocessing ablation, DPI-weighted optimization of the three strongest models, and spectral interpretability analysis using input gradients, SHAP attribution, and vegetation-index screening. This workflow was designed to evaluate not only whether wheat stem rust could be detected before visible symptoms appeared, but also which spectral regions supported the early predictions.

### 2.1 Dataset

We used a publicly available hyperspectral dataset of wheat stem rust described previously (Fedotov et al., 2025). The dataset is based on the spring wheat cultivar Saratovskaya 74, which is highly susceptible to *Puccinia graminis* f. sp. *tritici*. Seventy-five seeds were sown in each pot, and inoculation was carried out at 11 days after emergence, corresponding to BBCH growth stage 12. The controlled inoculation experiment produced time-series hyperspectral images of healthy and infected plants. Twelve pots are included in total, with six pots assigned to inoculation treatment and six serving as healthy controls.

Hyperspectral images were acquired indoors in a darkroom with a Cubert Ultris 20 snapshot camera. The spectral range was 450–874 nm, sampled in 106 bands at 4-nm intervals, and the spatial resolution was 410 × 410 pixels. Images were collected daily from 4 to 9 days post inoculation (DPI 4–9). Each day contributed 144 images, including 72 healthy and 72 infected samples, for a total of 864 images.

**Figure 2:**
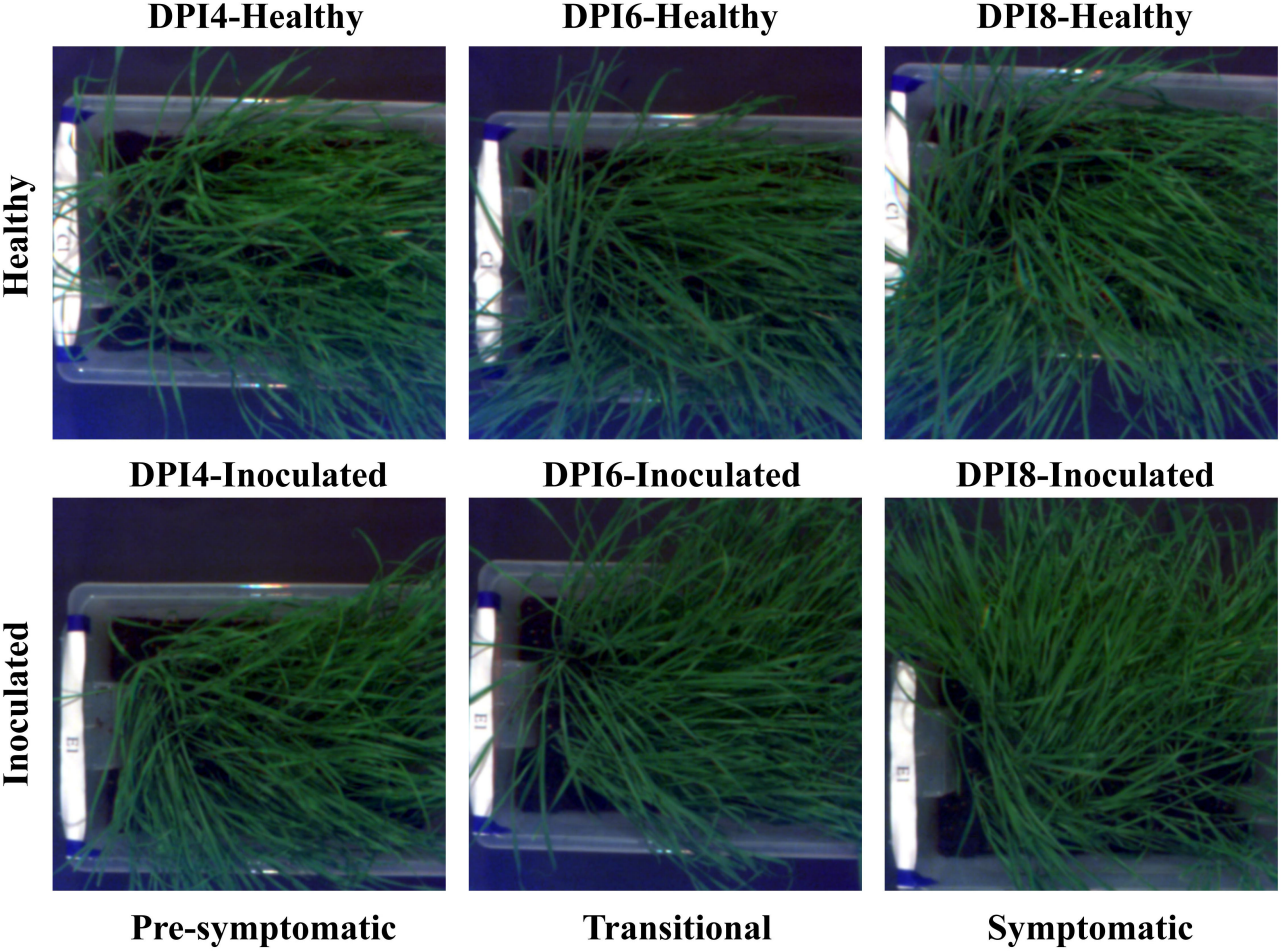
Hyperspectral true-color composite images of healthy and inoculated wheat plants at DPI 4, DPI 6, and DPI 8. The composites were generated using bands at 660 nm, 550 nm, and 470 nm as the red, green, and blue channels, respectively. The upper row shows healthy controls, and the lower row shows inoculated plants. Visual differences between the two groups remain limited at the early stage, indicating that pre-symptomatic infection is not readily distinguishable from overall appearance alone.

The dataset spans the key stages of stem rust development. DPI 4–5 corresponds to the pre-symptomatic stage, when plants still appear normal. DPI 6–7 marks a transitional stage with slight chlorosis. DPI 8–9 corresponds to the symptomatic stage, when rust-colored pustules are clearly visible. Representative true-color composites at DPI 4, DPI 6, and DPI 8 are shown in **Figure 2**. These examples illustrate the limited visual differences between healthy and inoculated plants at the early stage and the later emergence of obvious symptoms. This temporal structure makes the dataset suitable for evaluating model performance before symptom emergence.

### 2.2 Data partitioning

We adopted a pot-level stratified partitioning strategy so that different snapshots from the same pot did not appear in different subsets, thereby reducing the risk of label leakage (Luo et al., 2026). The dataset was split into 576 training images from 8 pots, 144 validation images from 2 pots, and 144 test images from 2 pots. Healthy and infected samples at each DPI were kept balanced within each subset. Pot-level partitioning was used instead of random image-level cross-validation because multiple snapshots from the same pot are visually and biologically correlated. This strategy reduced the risk of data leakage and provided a more conservative estimate of model performance.

### 2.3 Deep learning models

Seven deep learning models representing different architectural families were selected for systematic comparison (**Table 1**). The table summarizes their architecture types and core mechanisms. The selected models span basic convolutional networks, residual learning, attention mechanisms, 3D convolution, and hybrid architectures, which allows comparison of several feature-extraction strategies for hyperspectral pre-symptomatic detection. SimpleCNN follows a basic convolution-pooling-fully connected design (Chen et al., 2016). ResNet2D uses residual skip connections to alleviate degradation in deeper networks (He et al., 2016). Convolutional Block Attention Module (CBAM) adds channel and spatial attention (Woo et al., 2018). Spectral3DCNN uses 3D convolutions to learn joint spectral-spatial features (Li et al., 2017). HybridSN extracts spectral features with 3D convolutions and then spatial features with 2D convolutions (Roy et al., 2020). Spectral-Spatial Residual Network (SSRN) combines spectral and spatial residual blocks (Zhong et al., 2018). SpectralAttentionNet is a lightweight model designed in this study. Borrowing the channel-attention idea from Squeeze-and-Excitation networks (Hu et al., 2018), it assigns adaptive band weights through global average pooling and fully connected layers along the spectral dimension.

**Table 1:** Summary of the seven deep learning architectures.

Model	Architecture type	Core mechanism
SimpleCNN	2D convolution	Basic convolution-pooling-fully connected architecture
ResNet2D	2D residual network	Residual skip connections
CBAM	Attention mechanism	Dual channel-spatial attention
SpectralAttentionNet	Spectral attention	Adaptive weighting along the spectral dimension
Spectral3DCNN	3D convolution	Joint spectral-spatial feature extraction with 3D convolutions
HybridSN	Hybrid 3D + 2D architecture	3D spectral feature extraction followed by 2D spatial feature extraction
SSRN	Spectral-spatial residual network	Cascaded spectral and spatial residual blocks

All models were trained under a unified configuration. Inputs were MinMax-normalized to [0, 1] and then downsampled by 4× average pooling. Training used Adam with a learning rate of 1 × 10^-4^ and weight decay of 1 × 10^-5^. The batch size was 4, and the maximum number of epochs was 50 with early stopping (patience = 10). Each model was trained and tested with five random seeds (42, 123, 456, 789, and 1024). F1-score values are reported as mean ± standard deviation across repeated runs, whereas the remaining metrics are reported as averages.

### 2.4 Preprocessing ablation experiment

To assess the effect of preprocessing on deep learning performance, we designed three ablation settings using HybridSN. Because HybridSN extracts both spectral and spatial information and its 3D + 2D hybrid structure is broadly representative among the seven models, it was used as the baseline model for this analysis. The three settings were: (A) raw data with MinMax normalization; (B) NDVI masking for background removal; and (C) NDVI masking plus first-derivative transformation.

NDVI masking was used for background removal by calculating the normalized difference vegetation index, NDVI = (NIR − Red)/(NIR + Red), with the near-infrared (NIR) band set to 800 nm and the red band to 650 nm. A threshold of 0.3 was used to generate a binary mask. This threshold was used only for the preprocessing ablation experiment. Because NDVI masking produced only a modest improvement and raw data were retained as the standard input for the remaining model experiments, the main model comparison and DPI-weighted optimization did not depend on this threshold. The first-derivative transformation was calculated along the spectral dimension as the difference between adjacent bands, D_[i]_ = S_[i+1]_ − S_[i]_, converting the 106 original bands into 105 derivative features intended to emphasize rates of spectral change.

All ablation settings used the same training configuration. The F1-score was treated as the primary metric, and stability was assessed through repeated experiments with five random seeds.

### 2.5 DPI-weighted optimization strategy

DPI-wise analysis revealed a clear imbalance in performance across disease stages. Following the basic idea of Focal Loss (Lin et al., 2017)—namely, encouraging the model to pay more attention to difficult samples through loss reweighting—we designed a DPI-weighted optimization strategy. In this scheme, samples from the difficult stages (DPI 7–8) were given a loss weight between 1.0 and 1.5 by scaling their per-sample cross-entropy loss. This was intended to shift more learning toward these relatively difficult DPI stages. For the three best-performing models—HybridSN, SimpleCNN, and SpectralAttentionNet—we tested four settings, W = 1.0 (baseline), 1.2, 1.3, and 1.5. Each setting was evaluated with five random seeds.

### 2.6 Evaluation metrics

Classification performance was evaluated with F1-score as the primary metric, together with accuracy, precision, recall, and the area under the receiver operating characteristic curve (AUC). The F1-score, as the harmonic mean of precision and recall, is suitable for balanced binary classification. AUC was used to assess classification performance across different decision thresholds. The metric definitions are given in Equations 1–4 below.

(1) $$Precision=\frac{TP}{TP\;+FP}$$

(2) $$Recall=\frac{TP}{TP\;+FN}$$

(3) $$F1=\frac{2\times Precision\times Recall}{Precision+Recall}$$

(4) $$Accuracy=\frac{TP+TN}{TP+TN\;+FP\;+FN}$$

Here, TP, TN, FP, and FN denote true positives, true negatives, false positives, and false negatives, respectively. AUC was calculated as the area under the receiver operating characteristic (ROC) curve, which plots sensitivity against 1 − specificity across classification thresholds. All metrics range from 0 to 1, and higher values indicate better performance.

Particular attention was given to performance at the pre-symptomatic stage (DPI 4–5), as this is the most important indicator of practical usefulness. The above metrics were calculated separately for each DPI to quantify model behavior across disease stages.

### 2.7 Attribution analysis

To examine model decision patterns and identify key sensitive bands, we used three complementary approaches. First, we performed input gradient analysis on the original and DPI-weighted versions of SimpleCNN, SpectralAttentionNet, and HybridSN. By computing the absolute gradient of the target-class score with respect to each input band, this analysis captures local band-wise sensitivity. Second, we applied the GradientExplainer method in SHAP (SHapley Additive exPlanations) (Lundberg and Lee, 2017) to the best-performing 2D models for global attribution analysis. Third, we evaluated all 2–4-band combinations on NDVI-masked mean leaf spectra following a previously reported exhaustive vegetation-index screening framework (Deng et al., 2023a). This allowed us to check whether the key bands suggested by the models were also supported from a domain-knowledge perspective.

## 3 Results

### 3.1 Overall performance of deep learning models

**Figure 3:**
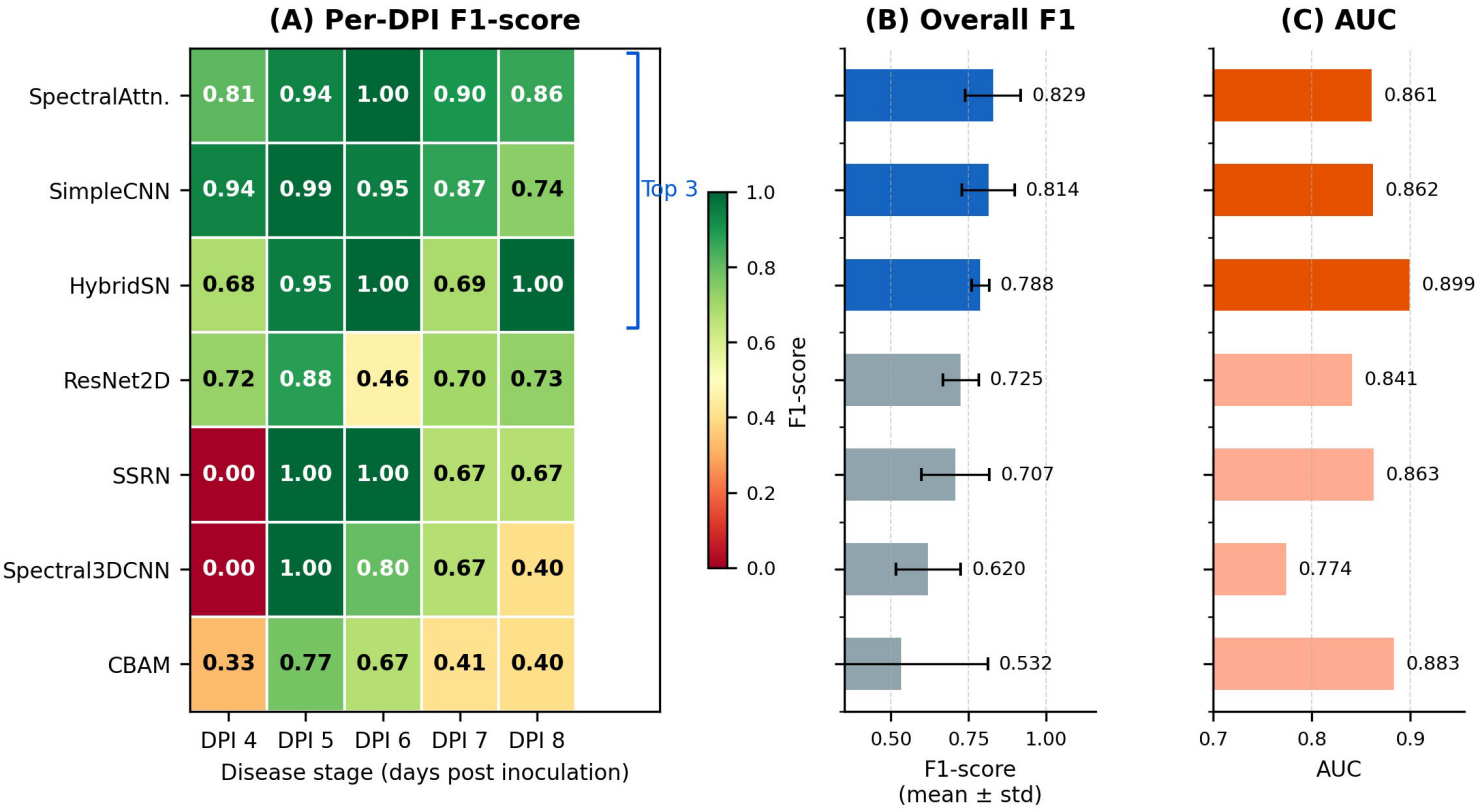
Multidimensional comparison of the seven deep learning models. **(A)** DPI-wise F1-score heatmap for DPI 4–8, with a white separator distinguishing the top three models from the remaining four models. **(B)** Overall F1-score shown as mean ± standard deviation. **(C)** AUC comparison across models.

**Table 2** summarizes the overall test performance of the seven deep learning models, and **Figure 3** provides a multidimensional comparison using DPI-wise F1-score patterns, overall F1-score, and AUC. F1-score values in **Table 2** are reported as mean ± standard deviation across five random seeds, whereas the other metrics are reported as averages. With 864 images in total, the top three models—SpectralAttentionNet, SimpleCNN, and HybridSN—reached F1-scores between 0.788 and 0.829, leaving only modest differences among them. The remaining four models—ResNet2D, SSRN, Spectral3DCNN, and CBAM—all had F1-scores below 0.73, indicating a clear drop in performance.

**Table 2:** Overall performance comparison of the seven deep learning models.

Rank	Model	F1-score (mean ± std)	Accuracy	Precision	Recall	AUC
1	SpectralAttentionNet	0.829 ± 0.089	81.9%	0.866	0.819	0.861
2	SimpleCNN	0.814 ± 0.085	80.3%	0.849	0.806	0.862
3	HybridSN	0.788 ± 0.028	79.4%	0.816	0.767	0.899
4	ResNet2D	0.725 ± 0.058	70.8%	0.706	0.792	0.841
5	SSRN	0.707 ± 0.110	73.5%	0.851	0.667	0.863
6	Spectral3DCNN	0.620 ± 0.105	68.3%	0.753	0.533	0.774
7	CBAM	0.532 ± 0.280	61.3%	0.552	0.633	0.883

F1-score values are reported as mean ± standard deviation across five random seeds; other metrics are reported as averages.

Model complexity was not positively related to performance. The simplest model, SimpleCNN (F1-score = 0.814), outperformed both ResNet2D (F1-score = 0.725), which uses residual connections, and CBAM (F1-score = 0.532), which uses dual attention. Under the present data scale, smaller models therefore appeared less prone to overfitting. HybridSN ranked third in overall F1-score (0.788) but had the smallest standard deviation (0.028), suggesting relatively stable behavior among the seven models. HybridSN also produced the highest AUC (0.899), although its relatively low recall (0.767) held back its F1-score.

The AUC ranking did not fully match the F1-score ranking. CBAM, for example, showed the lowest F1-score (0.532) but still achieved an AUC of 0.883, ranking fourth. This pattern suggests that threshold-dependent instability, rather than an absence of discriminative capacity, was the main factor behind its weak F1-score. AUC reflects ranking performance across thresholds, whereas F1-score depends on a fixed threshold. The difference between the two rankings therefore suggests that some models retained useful classification potential that was not fully reflected by F1-score alone.

Compared with the overall F1-score range of 0.86–0.94 reported for traditional machine learning with a systematic preprocessing pipeline on the publicly available dataset used in the present study (Fedotov et al., 2025), the unoptimized deep learning models did not show a clear overall advantage. After DPI-weighted optimization, however, the overall F1-scores of the top three models increased to 0.912–0.942, indicating that deep learning achieved an overall performance level comparable to that of the traditional approach on this dataset. This comparison should be interpreted as a benchmark-level reference, because the preprocessing pipeline, model family, and data partitioning strategy differed between the two studies.

### 3.2 DPI-wise detection performance

**Figure 4:**
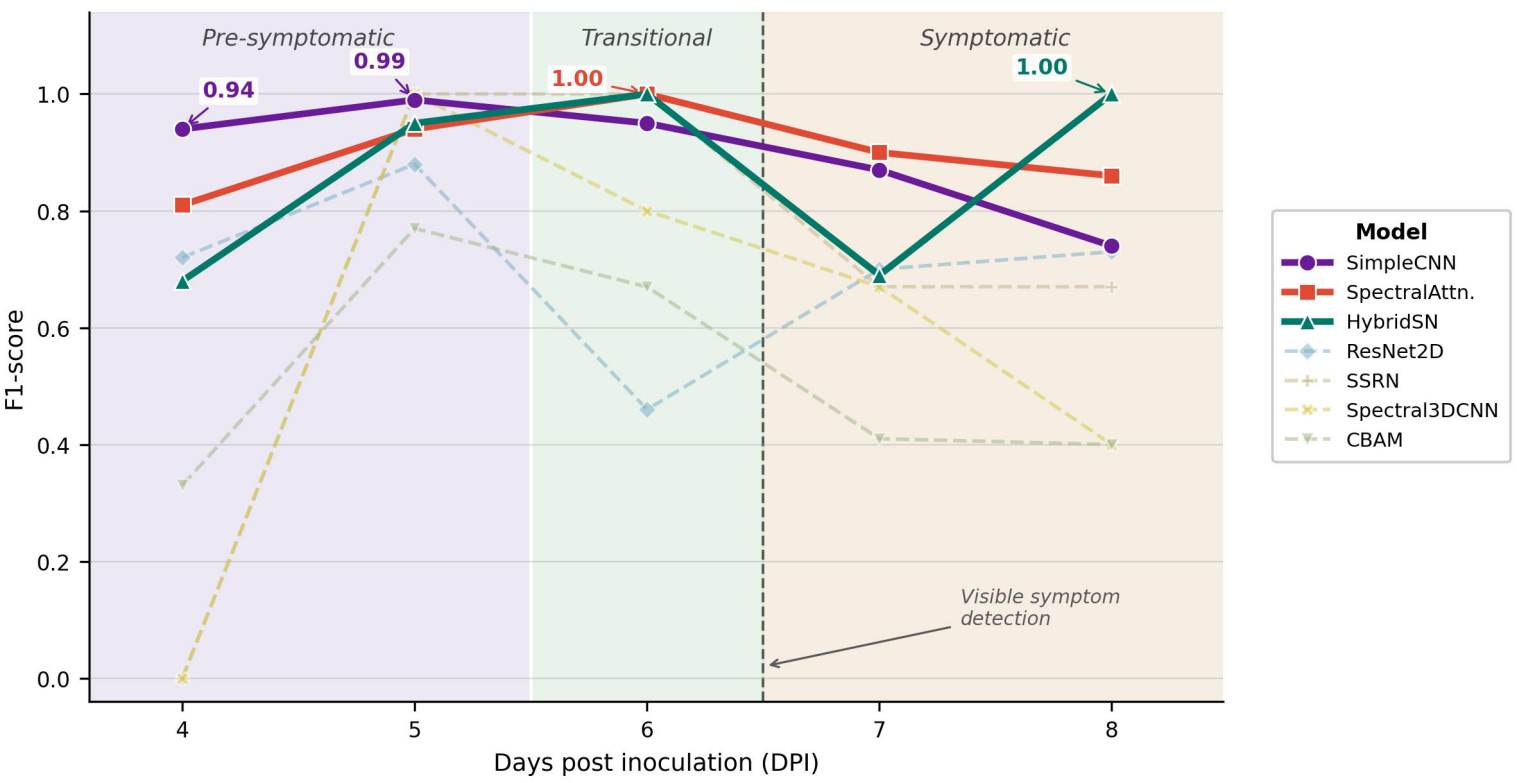
DPI-wise F1-score trends for the seven deep learning models from DPI 4 to DPI 8. Solid lines represent the three best-performing models overall, whereas dashed lines represent the remaining models. Background shading indicates the pre-symptomatic, transitional, and symptomatic stages, and the vertical dashed line marks the approximate timing of visible symptom detection at DPI 6–7.

To quantify detection performance across disease stages, we carried out a DPI-wise analysis for each model at DPI 4–8. DPI-wise F1-scores are reported in **Table 3** as mean ± standard deviation across five random seeds, with rounded mean values discussed in the text, and are visualized in **Figure 4**. The results are summarized in **Table 3** and visualized in **Figure 4**. Clear stage-dependent differences were observed among the models. DPI 4, the earliest pre-symptomatic stage with no visible symptoms, was the most challenging time point. SimpleCNN performed best at this stage (F1-score = 0.94), followed by SpectralAttentionNet (F1-score = 0.81), whereas SSRN and Spectral3DCNN failed to detect infected samples reliably at this stage. At DPI 5–6, disease-related spectral changes became more pronounced, and most models reached F1-scores of 0.95–1.00. At DPI 7–8, however, some models again showed noticeable fluctuations. This stage-specific instability motivated the later DPI-weighted optimization.

**Table 3:** DPI-wise F1-scores of the seven deep learning models (mean ± standard deviation across five random seeds).

Model	DPI 4	DPI 5	DPI 6	DPI 7	DPI 8
SpectralAttentionNet	0.805 ± 0.330	0.937 ± 0.130	1.000 ± 0.000	0.899 ± 0.130	0.858 ± 0.160
SimpleCNN	0.937 ± 0.130	0.992 ± 0.020	0.955 ± 0.090	0.871 ± 0.160	0.740 ± 0.250
HybridSN	0.685 ± 0.300	0.946 ± 0.110	1.000 ± 0.000	0.688 ± 0.040	1.000 ± 0.000
ResNet2D	0.717 ± 0.370	0.883 ± 0.150	0.457 ± 0.460	0.705 ± 0.080	0.733 ± 0.130
SSRN	0.000 ± 0.000	1.000 ± 0.000	1.000 ± 0.000	0.667 ± 0.370	0.667 ± 0.370
Spectral3DCNN	0.000 ± 0.000	1.000 ± 0.000	0.800 ± 0.400	0.667 ± 0.000	0.400 ± 0.490
CBAM	0.325 ± 0.410	0.771 ± 0.390	0.667 ± 0.370	0.408 ± 0.330	0.400 ± 0.330

Visible symptoms of stem rust usually begin with slight chlorosis at DPI 6, small lesions appear at DPI 6–7, and obvious pustules are seen at DPI 8 (Leonard and Szabo, 2005; Fedotov et al., 2025). In contrast, the deep learning models in this study detected the disease at DPI 4–5, before visible symptoms emerged. Compared with previously reported traditional machine-learning results (Fedotov et al., 2025), deep learning did not produce a much larger lead time overall. The clearest difference appeared at the most critical time point, DPI 4. Here, the best model in this study reached an F1-score of 0.94, above the reported upper bound of 0.83 for traditional machine learning. This points to stronger weak-signal recognition during the fully asymptomatic stage.

To further compare deep learning with the previously reported traditional machine-learning benchmark, we summarized the highest DPI-wise F1-scores from the present study and the corresponding upper-bound values reported by Fedotov et al. (2025) in **Table 4**. When each DPI was compared separately, deep learning outperformed the traditional benchmark at DPI 4, DPI 5, and DPI 8, with the clearest margin at DPI 4. At DPI 7, however, traditional machine learning still retained a small advantage. These results suggest that the main value of deep learning in this dataset lies less in replacing traditional methods across all stages than in improving recognition at the fully asymptomatic stage.

**Table 4:** Comparison of the highest DPI-wise F1-scores between the present study and traditional machine-learning results.

Source	DPI 4	DPI 5	DPI 6	DPI 7	DPI 8	Notes
Present study (DL, highest DPI-wise F1)	0.94	1.00	1.00	0.90	1.00	From **Table 3**
Traditional ML, upper bound	0.83	0.91	1.00	0.96	0.92	Compiled from the reported X^mean^/X^d1^ upper ranges in Fedotov et al. (2025)
Difference (DL − traditional ML)	+0.11	+0.09	0.00	−0.06	+0.08	Largest advantage at DPI 4

### 3.3 Preprocessing ablation experiment

**Table 5** presents the preprocessing ablation results for HybridSN. Relative to the raw-data baseline (F1-score = 0.788), NDVI masking produced a modest gain (F1-score = 0.808, + 2.6%), whereas adding first-derivative features on top of the mask reduced performance (F1-score = 0.751, −4.7%). This pattern contrasts with that reported for traditional machine learning on the publicly available dataset used in the present study (Fedotov et al., 2025). In that study, first-derivative transformation substantially improved the performance of traditional methods, whereas in our experiments the same operation reduced deep learning performance. Given the automatic feature-learning capacity of deep models and the limited gain from NDVI masking, raw data were retained as the standard input for the remaining experiments.

**Table 5:** Preprocessing ablation results for HybridSN.

Preprocessing strategy	F1-score	ΔF1
Raw data (MinMax normalization)	0.788 ± 0.028	Baseline
NDVI masking	0.808 ± 0.012	+0.020 (+2.6%)
NDVI masking + first derivative	0.751 ± 0.033	−0.037 (−4.7%)

### 3.4 DPI-weighted optimization

We applied the DPI-weighted optimization strategy to the top three models. Weight values of W = 1.0, 1.2, 1.3, and 1.5 were tested. Different models showed different weighting responses, but W = 1.5 provided the best overall balance across the three selected models when both overall F1-score and DPI-wise behavior were considered. In all three models, the weighted settings outperformed the baseline in overall F1-score, and the gain generally increased with the weight. Taking both overall F1-score and DPI-wise behavior into account, we selected W = 1.5 as the unified setting. Because stage-specific trade-offs were already observed in SimpleCNN and SpectralAttentionNet, larger weights were not further explored in this study. The corresponding overall results are summarized in **Table 6**.

**Table 6:** Results of DPI-weighted optimization.

Model	Baseline F1-score	Optimized F1-score	Improvement
SimpleCNN	0.814 ± 0.085	0.942 ± 0.080	+0.128 (+15.7%)
HybridSN	0.788 ± 0.028	0.933 ± 0.042	+0.145 (+18.4%)
SpectralAttentionNet	0.829 ± 0.089	0.912 ± 0.048	+0.083 (+10.0%)

Baseline values are the unweighted F1-scores reported in **Table 2**. Optimized values correspond to the DPI-weighted setting with W = 1.5. Improvement is reported as absolute F1-score change, with relative percentage change in parentheses.

The weighting strategy improved all three models. HybridSN showed the largest gain, increasing from 0.788 ± 0.028 to 0.933 ± 0.042 (+18.4%), whereas SimpleCNN achieved the highest optimized F1-score, increasing from 0.814 ± 0.085 to 0.942 ± 0.080 (+15.7%). SpectralAttentionNet also improved from 0.829 ± 0.089 to 0.912 ± 0.048 (+10.0%). These results indicate that DPI weighting improved overall classification performance without changing the model architectures.

**Figure 5:**
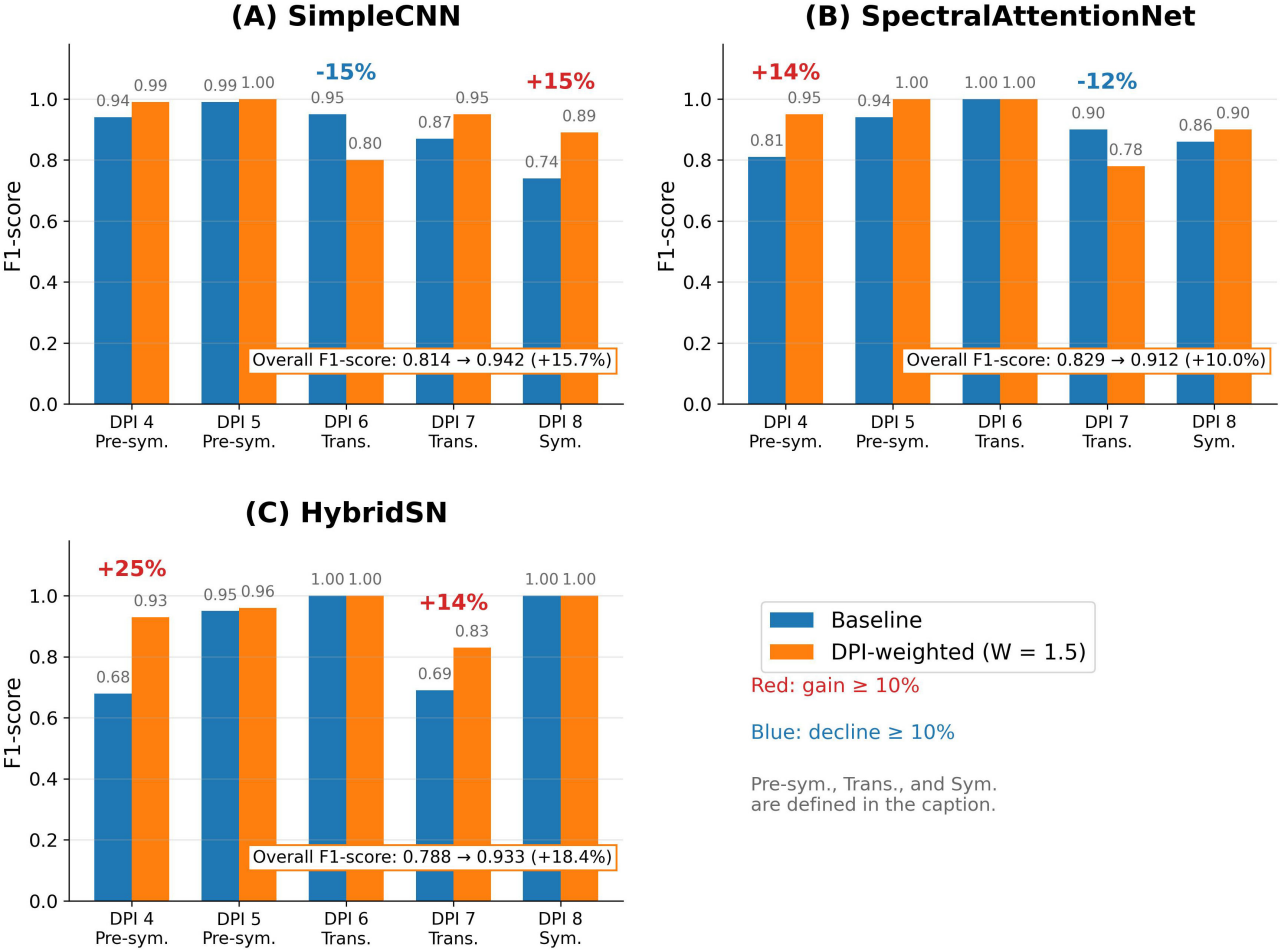
DPI-wise comparison of F1-scores before and after DPI-weighted optimization. **(A)** SimpleCNN. **(B)** SpectralAttentionNet. **(C)** HybridSN. Blue bars represent the baseline model, whereas orange bars represent the DPI-weighted model with W = 1.5. Red percentages indicate gains of at least 10%, and blue percentages indicate declines of at least 10%. The text box in each panel shows the change in overall F1-score.

DPI-wise changes before and after optimization are shown in **Figure 5**. The weighting strategy affected the three architectures differently. Notably, SimpleCNN reached an F1-score of 0.99 at DPI 4 after DPI-weighted optimization, representing the best pre-symptomatic detection result in this study. HybridSN improved or remained unchanged at all five DPIs, indicating the most stable DPI-wise response. SimpleCNN and SpectralAttentionNet, by contrast, both gained in overall F1-score but showed local declines at specific stages, namely DPI 6 for SimpleCNN and DPI 7 for SpectralAttentionNet. This pattern suggests that DPI weighting improved overall performance but could introduce stage-specific trade-offs in some architectures.

Because HybridSN showed the largest overall gain and the most stable DPI-wise response, its stage-specific results are further summarized in **Table 7**. After optimization, HybridSN improved from 0.68 to 0.93 at DPI 4 and from 0.69 to 0.83 at DPI 7. These results show that DPI weighting can steer the model toward difficult samples and improve performance at the pre-symptomatic and transitional stages without increasing the amount of training data.

**Table 7:** DPI-wise performance of HybridSN before and after optimization.

DPI	Stage	Baseline F1	Optimized F1	Improvement
4	Pre-symptomatic	0.68	0.93	+0.25 (+37%)
5	Pre-symptomatic	0.95	0.96	+0.01 (+1%)
6	Transitional	1.00	1.00	0.00
7	Transitional	0.69	0.83	+20%
8	Symptomatic	1.00	1.00	0.00

## 4 Discussion

### 4.1 Realization and significance of pre-symptomatic detection

Combining deep learning with hyperspectral imaging made it possible to detect wheat stem rust at the earliest pre-symptomatic stage (DPI 4), when no visible symptoms were present, with F1-scores of 0.93–0.99. This was earlier than the appearance of visible symptoms at DPI 6–7. In practical terms, such earlier detection matters. Curative fungicide application within 1–5 days after inoculation can still be effective, whereas efficacy declines markedly after 7 days (Schmitt et al., 2025). Earlier identification may therefore create a useful window for intervention, whether for fungicide application or for quarantine-type measures. Comparable pre-symptomatic detection has also been reported in other crop-disease systems (Nguyen et al., 2021; Cao et al., 2022).

### 4.2 Model selection and the challenge of limited data

Under limited-data conditions, SimpleCNN consistently showed the strongest detection performance at DPI 4 (baseline F1-score = 0.94; optimized F1-score = 0.99). This suggests that with only 864 images, a simpler architecture with fewer parameters was less prone to overfitting than more complex models. HybridSN ranked third by baseline F1-score (0.788), yet it benefited most from weighting (+18.4%), improving from 0.68 to 0.93 at DPI 4. This aligns with its high AUC (0.899), which suggests that the model retained useful discriminative capacity that was not fully expressed at the original operating point. A similar gap between F1-score and AUC also appeared for CBAM (F1-score = 0.532, AUC = 0.883), again pointing to threshold-dependent performance rather than architecture alone as a main source of weak performance.

The DPI-wise trends in **Figure 4** suggest an interaction between spectral signal strength and architectural complexity. At DPI 5–6, disease-related spectral changes had become strong enough that most models achieved F1-scores of at least 0.77 (**Table 3**), and differences among architectures narrowed. When the signal was stronger, architecture choice mattered less. At the weakest-signal stage, DPI 4, the models diverged sharply (F1-score range: 0.00–0.94). SimpleCNN, with relatively few parameters, still detected disease at DPI 4 (F1-score = 0.94), whereas SSRN and Spectral3DCNN failed at that stage (F1-score = 0.00). Both recovered to F1-score = 1.00 at DPI 5, which suggests that the DPI 4 failure reflected difficulty fitting weak signals under limited data rather than a basic architectural defect. SimpleCNN and HybridSN also followed opposite trajectories over disease progression: SimpleCNN performed best at DPI 4–5 (F1-score = 0.94–0.99) but declined to 0.74 at DPI 8, whereas HybridSN started at 0.68 at DPI 4 and rose to 1.00 at DPI 8. Together with the input-gradient results in Section 4.3, this pattern points to different feature preferences across architectures. SimpleCNN treats spectral bands as channels and integrates them jointly with 2D convolutions, which may make it better suited to weak global variation in the 480–550 nm blue–green region during the pre-symptomatic stage. HybridSN, in contrast, models spectral information explicitly with 3D convolutions and appears more responsive to stronger signals in the 580–680 nm red region during the symptomatic stage. This also helps explain the role of DPI weighting: by changing the contribution of samples from different stages, it redistributes attention toward signals that are otherwise underused.

### 4.3 Exploration of spectral interpretability

To better understand model behavior and to provide guidance for future sensor design, we examined band importance from three angles: model-driven analysis (input gradients), global attribution (SHAP), and domain-knowledge-based screening with vegetation indices.

#### 4.3.1 Input gradient analysis

**Figure 6:**
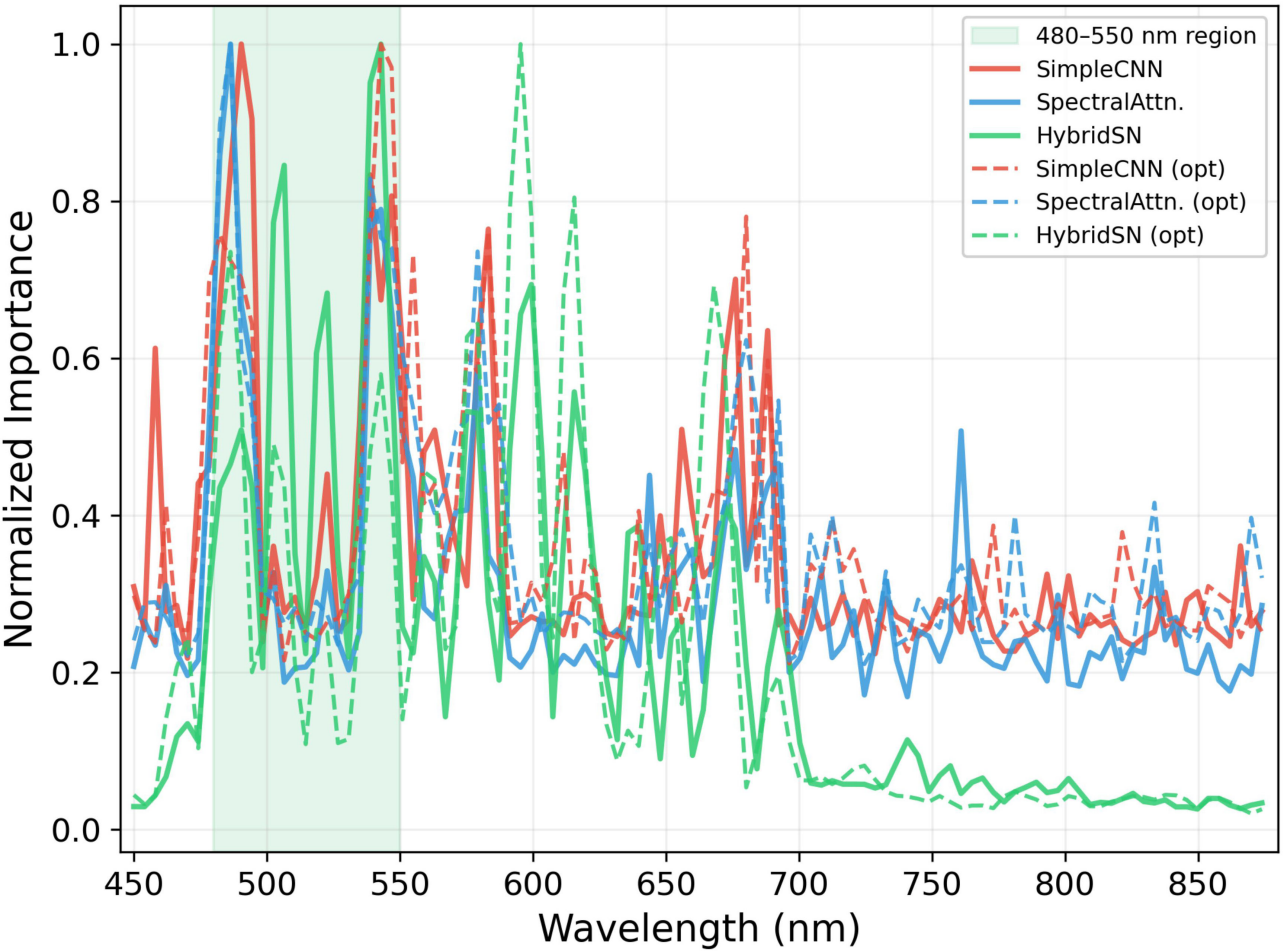
Band-importance distributions from input-gradient analysis before and after DPI-weighted optimization. Solid lines represent baseline models, whereas dashed lines represent DPI-weighted models. The green shaded region indicates the 480–550 nm chlorophyll-sensitive interval. Gradient values were normalized to the maximum value of each model to facilitate comparison across models.

From a model-driven perspective, we performed input gradient analysis for the original and DPI-weighted versions of Simple CNN, SpectralAttentionNet, and HybridSN (Simonyan et al., 2014). This method calculates the absolute gradient of the target-class score with respect to each input band and therefore provides a direct view of local band-wise sensitivity. The top five bands identified for each model are summarized in **Table 8**, and the corresponding band-importance distributions are shown in **Figure 6**.

**Table 8:** Top five bands identified by input-gradient analysis for each model.

Model	Optimization status	1	2	3	4	5
SimpleCNN	Baseline	490	494	486	539	547
SpectralAttentionNet	Baseline	486	482	543	539	490
HybridSN	Baseline	543	539	507	503	599
SimpleCNN	DPI-weighted	543	547	680	539	583
SpectralAttentionNet	DPI-weighted	486	482	539	543	547
HybridSN	DPI-weighted	595	616	591	599	486

Across the six model variants, most of the strongest responses were concentrated in the 480–550 nm region. Five of the six variants placed bands in the 530–550 nm range within their top five, and the 480–495 nm blue region also remained important in most cases. This suggests that pre-symptomatic recognition depended strongly on the blue–green region associated with chlorophyll-related metabolism.

HybridSN with DPI weighting was the main exception. Its top five bands shifted toward the 595–616 nm red region, although 486 nm remained among the top five. This shift suggests that the interaction between DPI weighting and 3D convolution made the model more responsive to stronger red-region signals. The same region is broadly consistent with the feature-importance pattern reported for a first-derivative model on the same dataset, in which the red region contributed strongly (Fedotov et al., 2025). By contrast, the weighted SimpleCNN still responded to bands in the blue–green, red, and near-infrared regions, indicating a more mixed use of spectral information.

**Figure 7:**
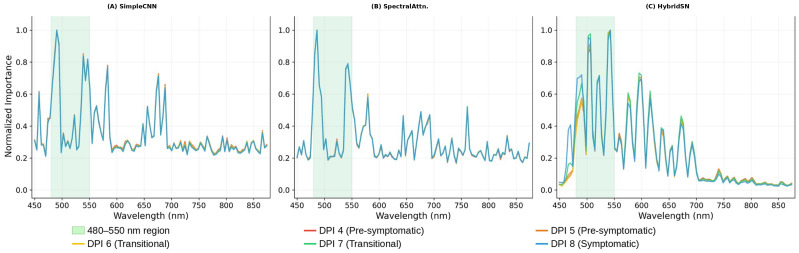
DPI-wise band-importance distributions from input-gradient analysis. **(A)** SimpleCNN. **(B)** SpectralAttentionNet. **(C)** HybridSN. The green shaded region indicates the 480–550 nm chlorophyll-sensitive interval. Gradient values at each DPI were normalized separately to facilitate comparison of relative importance patterns across stages.

DPI-wise input-gradient patterns further revealed architectural differences across disease progression (**Figure 7**). SimpleCNN and SpectralAttentionNet showed highly similar band-importance patterns from DPI 4 to DPI 8, suggesting that the two 2D models relied on broadly similar extraction strategies across stages. HybridSN, by contrast, showed clearer separation between DPIs: at DPI 7–8, responses in both the 480–550 nm and 580–680 nm regions were stronger than at DPI 4–6. Despite these differences, all three models retained major peaks in the 480–550 nm range, suggesting that chlorophyll-related spectral variation remained important throughout disease development.

#### 4.3.2 Exhaustive screening of vegetation indices

From a domain-knowledge perspective, we screened all 2–4-band combinations on NDVI-masked mean leaf spectra following a previously reported exhaustive vegetation-index framework (Deng et al., 2023a). The main results are summarized in **Table 9**. Because many band combinations were tested, this screening analysis should be regarded as exploratory. The selected band combinations were therefore used as supporting spectral evidence rather than as definitive physiological biomarkers. The screening results placed 530.8 nm at the core of both the best two-band and best three-band combinations. The best four-band index, GARI, and the second-ranked four-band index, EMBI, likewise pointed to the importance of the green region.

**Table 9:** Results of exhaustive vegetation-index screening.

Band number	Rank	Best index	Band combination (nm)	F1
2	1	MGRVI	530.8, 542.9	0.900
3	1	ARI2	530.8, 575.2, 631.7	0.927
4	1	GARI	546.9, 587.3, 660.0, 684.2	0.933
4	2	EMBI	526.7, 530.8, 575.2, 639.8	0.929

MGRVI, modified green-red vegetation index; ARI2, anthocyanin reflectance index 2; GARI, green atmospherically resistant vegetation index; EMBI, enhanced modified bare soil index.

The model-driven and domain-knowledge-driven analyses both pointed to the 480–550 nm blue–green region, although not in exactly the same way. Vegetation-index screening pinpointed 530.8 nm more precisely: it occupied a central position in the best two-band and three-band combinations and also appeared in the second-ranked four-band EMBI index. The deep learning models, by contrast, drew on a broader interval from 480 to 550 nm rather than on one or two isolated bands. This suggests that the models used coordinated information across neighboring bands. The finding is in line with plant physiology, as early pathogen infection can alter chlorophyll synthesis and defence-related pigments, both of which are expressed strongly in this part of the spectrum (Wan et al., 2022). A similar emphasis on green bands was also reported for the mean-curve model in a traditional machine-learning study on the same dataset (Fedotov et al., 2025).

From a cross-disease perspective, the optimal MGRVI and ARI2 combinations identified here were highly consistent with wheat stripe rust results centered on bands near 531 nm (Deng et al., 2023a). More broadly, this spectral region has appeared repeatedly in other crop-disease studies, including rice bacterial leaf blight, wheat powdery mildew, and rice leaf blast (Khan et al., 2021; Tian et al., 2021; Cao et al., 2022). Although these studies involve different crops and diseases, together they reinforce the broader importance of the green region for early disease response.

#### 4.3.3 Global SHAP attribution analysis

**Figure 8:**
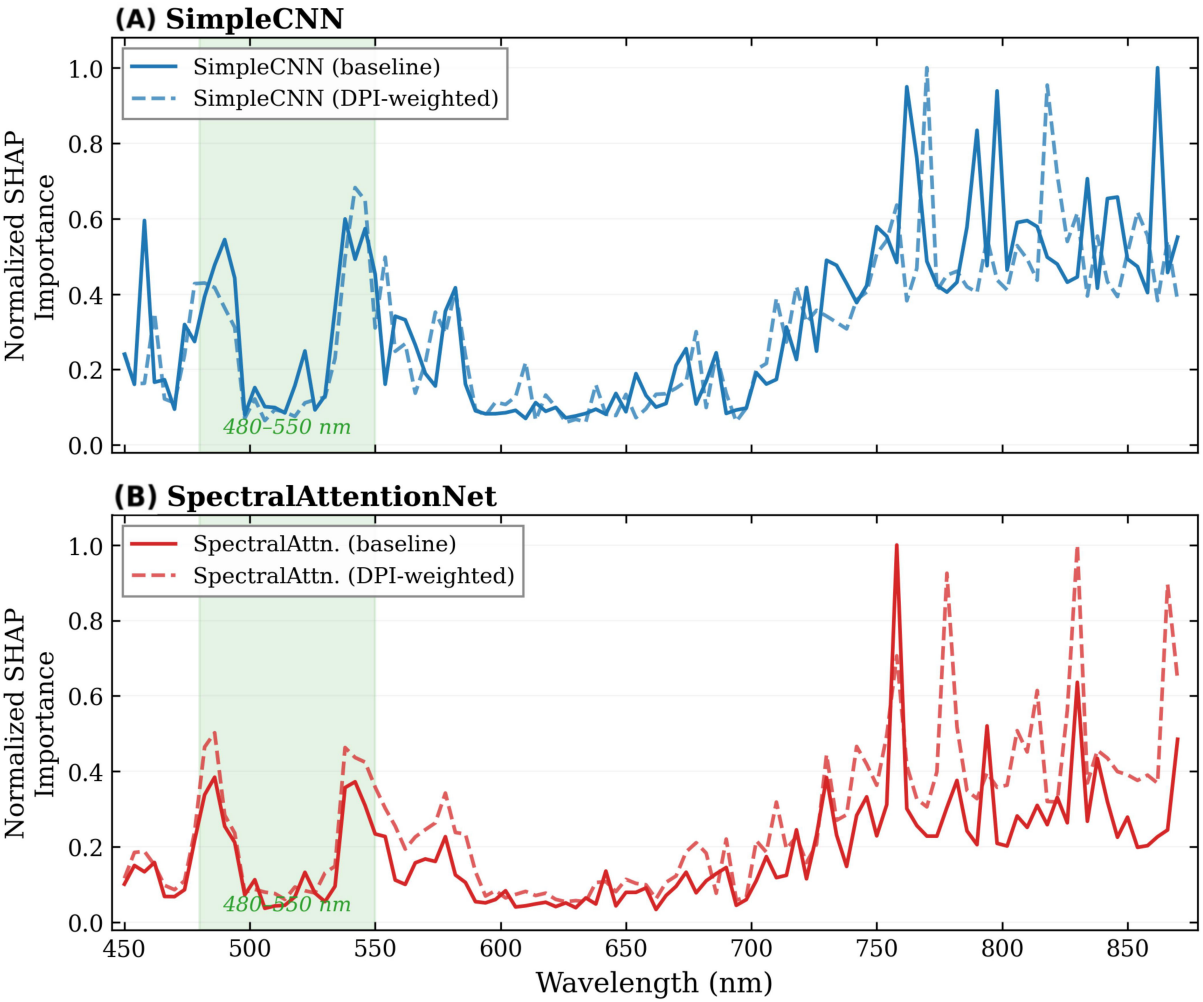
SHAP-based band-importance distributions for SimpleCNN and SpectralAttentionNet before and after DPI-weighted optimization. Solid lines denote baseline models, whereas dashed lines denote DPI-weighted models. The green shaded region indicates the 480–550 nm chlorophyll-sensitive interval. SHAP values were normalized to the maximum value of each model to facilitate comparison across models.

To test whether the input-gradient results were robust, we further applied SHAP GradientExplainer (SHapley Additive exPlanations) (Lundberg and Lee, 2017) to SimpleCNN and SpectralAttentionNet, including both baseline and DPI-weighted versions. Fifty training images were sampled at random as the background set, and SHAP values were then calculated for each test image. Absolute SHAP values were averaged across spatial dimensions to obtain band-level attribution scores. HybridSN was not included in this analysis because the memory demand of its 3D convolutional feature maps was too high for GradientExplainer under the available hardware conditions. The top five SHAP bands are summarized in **Table 10**, and the corresponding band-importance distributions are shown in **Figure 8**.

**Table 10:** Top five bands identified by global SHAP attribution.

Model	Version	1	2	3	4	5	480–550 nm (%)
SimpleCNN	Baseline	862	762	798	790	766	15.7
SimpleCNN	DPI-weighted	770	818	822	542	546	14.6
SpectralAttentionNet	Baseline	758	830	794	870	838	16.1
SpectralAttentionNet	DPI-weighted	830	778	866	758	870	14.4

SHAP analysis provided information that complemented the input-gradient results. Across all four model variants, the top five SHAP bands were concentrated mainly in the 750–870 nm near-infrared region rather than in the 480–550 nm blue–green region emphasized by the input gradients. This suggests that the near-infrared range contributed more general evidence of disease presence, whereas the blue–green region was more strongly associated with local sensitivity in early classification decisions.

This separation between sensitivity and contribution has a methodological implication. Input gradients reflect local perturbation sensitivity, whereas SHAP measures global marginal contribution. Near-infrared reflectance is strongly shaped by changes in internal leaf structure and therefore dominated the absolute attribution scores. By contrast, although the blue–green region has lower absolute reflectance, it is sensitive to early shifts in chlorophyll-related metabolism. This contrast is consistent with reports that near-infrared reflectance increases during the latent period of stripe rust (Liu et al., 2015).

SimpleCNN with DPI weighting was the only model whose top five SHAP bands included both core green bands (542–546 nm) and near-infrared bands. This joint use of metabolic and structural signals is consistent with its strong performance at DPI 4 (F1-score = 0.99). At the same time, the 480–550 nm interval accounted for 14.4%–16.1% of the total SHAP attribution across all 106 bands, close to its share of the spectral channels themselves (17.0%). In other words, this region did not disappear in the global attribution view; it simply did not dominate in the same way as it did in the sensitivity analysis.

**Figure 9:**
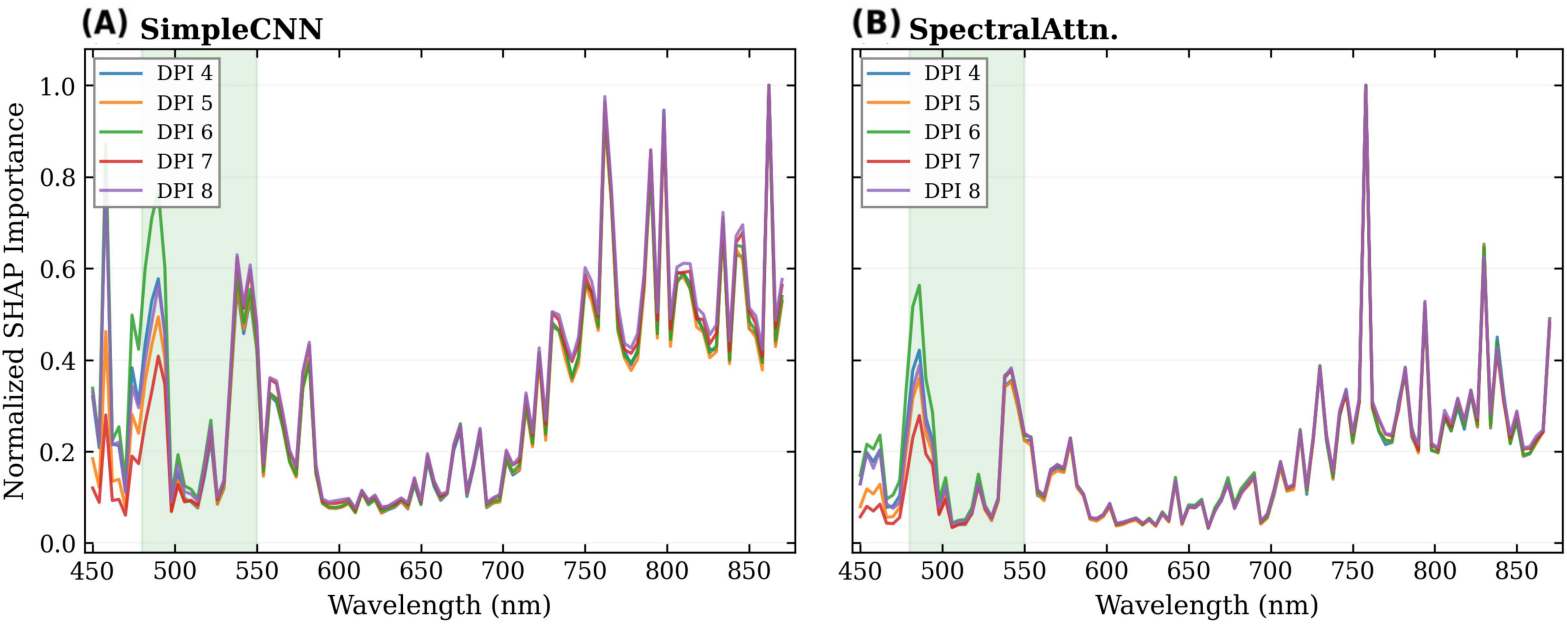
DPI-wise SHAP-based band-importance distributions for the baseline 2D models. **(A)** SimpleCNN. **(B)** SpectralAttentionNet. The green shaded region indicates the 480–550 nm chlorophyll-sensitive interval. SHAP values at each DPI were normalized separately to facilitate comparison of relative importance patterns across stages.

DPI-wise SHAP analysis for the baseline 2D models is shown in **Figure 9**. SimpleCNN and SpectralAttentionNet maintained highly consistent band-attribution distributions across disease stages, which agrees with the input-gradient result that these models relied on similar feature strategies across DPIs. In SpectralAttentionNet, the top five bands at DPI 6 included 482 and 486 nm, which may reflect the period at which chlorophyll-related change began to accumulate more rapidly.

#### 4.3.4 Integrated analysis

Taken together, the input-gradient analysis, SHAP-based global attribution, and exhaustive vegetation-index screening all point to the 480–550 nm blue–green region as central to pre-symptomatic detection by the deep learning models, whereas the 750–870 nm near-infrared region appears to contribute more general evidence of disease presence. The former is mainly associated with chlorophyll and related pigments, whereas the latter reflects structural change in leaves. Earlier recognition seems to depend on both types of information.

It should also be noted that both input gradients and SHAP are *post hoc* interpretation methods based on gradient information and therefore still carry limitations, including sensitivity to noise and reliance on local linear approximation. More robust approaches, such as Integrated Gradients, could be used for cross-checking in future work.

### 4.4 Complementarity between deep learning and traditional methods

Traditional machine learning and deep learning each have their own place in hyperspectral disease detection. A carefully designed preprocessing pipeline raised the F1-score of traditional machine learning from 0.67–0.75 to 0.86–0.94 on the publicly available dataset from Fedotov et al. (2025), a result that is broadly comparable to that of the DPI-weighted deep learning models reported here (F1-score = 0.912–0.942). A DPI-wise comparison nevertheless shows that the clearest difference lies in the earliest asymptomatic stage. At DPI 4, the best traditional machine-learning result was 0.83, whereas the highest deep-learning result in this study reached 0.94. At DPI 7, by contrast, traditional machine learning still retained a small advantage. Deep learning therefore appears less important as a full replacement for traditional methods than as a way of improving recognition during the weakest-signal stage while reducing dependence on hand-crafted features. The same overall pattern is consistent with earlier work on hyperspectral disease detection under symptomatic versus asymptomatic conditions (Khan et al., 2021; Tian et al., 2021).

The preprocessing ablation experiment supports this difference at the methodological level. In traditional machine learning on the publicly available dataset from Fedotov et al. (2025), first-derivative transformation was crucial, whereas in our experiments the same operation reduced deep learning performance by 4.7%. A similar contrast has also been noted in near-infrared spectral analysis (Zhang et al., 2019), and second-derivative transformation has likewise been shown to improve traditional discriminative models (Liu et al., 2015). Deep learning, in contrast, relies more heavily on end-to-end representation learning and therefore depends less on hand-designed spectral transformations. In this sense, traditional machine learning remains attractive for its interpretability and lower computational cost, whereas deep learning appears to offer more room for early weak-signal detection.

### 4.5 Limitations and future perspectives

The main limitation of this study is the narrow data source. All 864 images came from the same cultivar (Saratovskaya 74), the same environment (an indoor darkroom), and the same inoculation protocol. The spectral patterns learned by the models may therefore be closely tied to the physiological response of that particular cultivar. Under field conditions, cultivars can differ in resistance genes and spectral response, and detection may also be affected by natural illumination, soil background, and mixed infection. Whether the present findings will generalize to more complex field conditions therefore still needs to be tested on larger datasets that cover multiple cultivars and environments. In addition, the study did not include mixed diseases or abiotic stress, used one unified hyperparameter setting for all models, and did not evaluate Transformer-based architectures.

The most important direction for future work is to expand data collection under field conditions. This includes adding samples from multiple cultivars and growth conditions to test generalization, and introducing natural illumination and soil background variation to assess robustness. Preliminary evidence for cross-crop transfer is already available, and the performance gap between laboratory studies and field deployment has likewise been noted elsewhere (Shafay et al., 2025; Terentev et al., 2025). The key bands identified here, especially those in the 480–550 nm region, may also be useful when choosing bands for multispectral sensor design and may help reduce the cost of field-deployable systems. Finally, the DPI-weighted strategy is simple and effective in the present setting, but its value in other crop-disease systems still needs to be tested.

## 5 Conclusion

Using a hyperspectral imaging dataset, this study systematically evaluated the potential of deep learning for pre-symptomatic detection of wheat stem rust. The main conclusions are as follows.

1. Deep learning can support pre-symptomatic detection. At DPI 4, when no visible symptoms were present, the three best-performing optimized models all achieved F1-scores of at least 0.93, and SimpleCNN reached 0.99. These results show that, under controlled indoor conditions, hyperspectral imaging combined with deep learning can identify stem rust before visible symptoms develop.
2. The models achieved reliable detection at 4 days post inoculation, earlier than the onset of visible symptoms at DPI 6–7. Deep learning can therefore move detection forward to the pre-symptomatic stage and create a useful window for disease management.
3. The DPI-weighted optimization strategy effectively improved detection at difficult stages. Across the three models, overall F1-score gains ranged from +10.0% to +18.4%, with HybridSN showing the largest gain.
4. Input gradient analysis, SHAP-based global attribution, and vegetation-index screening all pointed to the 480–550 nm blue–green region as the key spectral interval for pre-symptomatic detection, whereas the near-infrared region (750–870 nm) contributed more general information related to disease presence.

## Data Availability

Publicly available datasets were analyzed in this study. This data can be found here: The dataset analyzed in this study is publicly available in the online repository reported by Fedotov et al. (2025). The data can be accessed at: https://drive.google.com/drive/folders/1vpKPlPw5uK5AnKctaE2oYCuOaRFX4-yN.
